# Evaluation of muco-adhesive tacrolimus patch on caspase-3 induced apoptosis in oral lichen planus: a randomized clinical trial

**DOI:** 10.1186/s12903-023-02803-8

**Published:** 2023-02-14

**Authors:** Suzan S. Ibrahim, Nivine I. Ragy, Noha A. Nagy, Hala El-kammar, Asmaa M. Elbakry, Ola M. Ezzatt

**Affiliations:** 1 Department of Oral Medicine, Periodontology and Oral Diagnosis, Faculty of Dentistry, Ain Shams University, Cairo, Egypt; 2 Department of Oral Medicine, Periodontology Oral Diagnosis and Radiology, Faculty of Dental Medicine, Nahda University, Beni Suef, Egypt; 3 Department of Oral Medicine, Periodontology, and Oral Diagnosis, Faculty of Oral and Dental Medicine, Future University in Egypt, St. South 90Th, New Cairo 1, Cairo, 11835 Egypt; 4 Department of Oral Pathology, Faculty of Oral and Dental Medicine, Future University in Egypt, Cairo, Egypt; 5 Department of Pharmaceutics and Pharmaceutical Technology, Faculty of Pharmacy, Al-Azhar University, Cairo, Egypt; 6 Department of Pharmaceutics and Pharmaceutical Technology, Faculty of Pharmacy, Heliopolis University, Cairo, Egypt; 7 Department of Oral Medicine, Periodontology and Oral Diagnosis, Faculty of Dentistry, Ain Shams University, 20 Organization of African Union St., Cairo, 1156 Egypt

**Keywords:** Apoptotic keratinocytes, Caspase-3, Oral lichen planus, Tacrolimus, Triamcinolone acetonide

## Abstract

**Background:**

The study compared the clinical effectiveness of topical Tacrolimus (TAC) in patches or gel with Triamcinolone acetonide (TRI) gel for erosive/atrophic oral lichen planus (OLP) and investigated the influence of these therapies on Caspase-3 expression as a marker of apoptosis.

**Methods:**

Thirty patients were randomly assigned into three equal groups to receive either topical TAC 0.1% patch twice daily, topical TAC 0.1% gel, or topical TRI 0.1% gel four times daily for 8 weeks. Each patient's clinical score (CS), visual analogue scale (VAS), and total atrophic area (TAA) of the marker lesion were measured at baseline, 2, 4, and 8 weeks of treatment, as well as after 4 weeks of treatment free period. Caspase-3 expression and lymphocytic counts (LC) were assessed in pre- and post-treatment biopsied stained sections.

**Results:**

TAC patch resulted in a higher reduction in CS [− 14.00 (15.54%)] and VAS [− 70.21 (15.82%)] followed by TAC gel then TRI gel within the first two weeks. The reduction in VAS and TAA were significantly higher in TAC groups compared to TRI gel, although the difference between TAC treatment was not significant and this was observed throughout the treatment and follow-up periods. Caspase-3 expression increased in connective tissue in all groups. It decreased significantly within the epithelium in both TAC groups but increased in TRI gel. (LC) were significantly lowered with the TAC patch compared to other groups. The percentage change in Caspase-3 epithelial expression was significantly correlated to the CS, TAA, and LC.

**Conclusion:**

Both TAC patch and gel significantly decreased pain and lesion size than TRI gel, with a significant reduction in Caspase-3 expression within the epithelium in comparison to the increase seen with TRI gel. The study protocol was registered at www.clinicaltrials.gov (NCT05139667) on 01/12/2021.

**Supplementary Information:**

The online version contains supplementary material available at 10.1186/s12903-023-02803-8.

## Introduction

Oral lichen planus (OLP) is a chronic inflammatory T-cell-mediated disorder that affects the oral mucosa, with an estimated general population prevalence of 0.89% [1]. There are six types of OLP lesions: reticular, papular, plaque-like-white forms, and the symptomatic atrophic (erythematous), erosive (ulcerated), and bullous-red forms, which may occur alone or in various combinations [2].

Microscopically, OLP is characterized by dense subepithelial lymphocytic infiltrate and degeneration of basal keratinocytes, as well as apoptotic keratinocytes seen by light microscope as shrunken cells with condensed nuclei and eosinophilic cytoplasm (Civatte/Colloid bodies) [3]. Apoptosis in OLP is induced by P53, B-cell lymphoma-2 (BCL-2) family proteins, Fas/FasL pathway, proteases of the matrix metalloproteinase-9 (MMP-9) and caspase-3 [4].

Caspases are cysteine-proteases required for programmed cell death and activated by two pathways; the extrinsic pathway, which is triggered by attachment of “death receptors” [TNF] family, TNF-1 and FasL on the cell surface, while the intrinsic pathway is triggered by various forms of stress, including inadequate cytokine support and different types of intracellular damage [5]. Caspase-3 (CPP32) is the most downstream enzyme in the apoptosis-inducing protease pathway and is clearly associated with cell death in OLP lesions [6].

Different therapies have been developed to control symptomatic OLP, but a permanent cure is not yet available. Although many reviews on OLP therapy recommend topical steroids as the first-line treatment for symptomatic OLP, many other therapies represented effective alternatives for its management but required further research [7].

Calcineurin inhibitors are immunomodulators that bind to intracytoplasmic proteins in T-lymphocytes (cyclosporine to cyclophilin; tacrolimus and pimecrolimus to FK506-binding protein), which in turn inhibit calcineurin, leading to suppression of transcription and production of variable cytokines and suggesting a possible role of these agents in management of variable immune-mediated lesions [8].

Clinical trials with calcineurin inhibitors in the treatment of symptomatic OLP have yielded promising results [9–11]. Furthermore, recent systematic reviews and meta-analysis have concluded that topical tacrolimus is a safe and effective alternative to topical corticosteroids for OLP treatment [12, 13].

The use of currently available topical formulations has a few limitations like reduced patient compliance and salivary washout. In contrast, muco-adhesive buccal film provides the advantage of advanced retention time, increased patient compliance, prolonged release of the drug and better bioavailability at the site of action [14]. To the best of the author’s knowledge, there is one published in vitro and in vivo study the investigated the use of muco-adhesive tacrolimus patches for oral mucosal delivery as a drug model conducted on goat mucosa and showed promising results [15].

We hypothesized that tacrolimus delivered via a mucoadhesive patch would improve clinical outcomes and patient compliance more than tacrolimus or triamcinolone acetonide topical gel. Thus, this study aimed to evaluate the clinical efficacy of tacrolimus 0.1% in mucoadhesive patches compared to tacrolimus or corticosteroids in gel form for symptomatic oral lichen planus and to investigate the effect of these medications on the expression of caspase 3 in oral lichen planus lesions as an early marker of apoptosis.

## Materials and methods

### Sample size calculation

We postulated that if the clinical score (CS) dropped a minimum of 10%, 20%, or 25% with corticosteroid gel, tacrolimus gel and tacrolimus patch, respectively, indicating that 10–15% difference in CS between groups would mean clinically significant results based on the outcome of previous clinical trials [16, 17]. A decrease of this size with a two tailed p-value of 0.05 and power of 0.80 would require a total sample size of 26 patients. Allowing for a non-adherence rate of 10%, we assume that 30 patients will be recruited for this study to compensate for drop outs (10 per treatment group), using G-power analysis program (G*Power version 3.1.9.7).

### Study design, randomization, and blinding

This study was a prospective, three-parallel arms, assessor-blinded, 12-week randomized clinical trial that included 30 patients diagnosed with erosive or atrophic OLP lesions. Eligible patients were randomized in a 1:1:1 ratio using computer-generated randomization assignment into three groups. The allocation concealment group sealed envelopes by a non-involved investigator. The outcome assessors were blinded to the type of intervention used.

### Recruitment and eligibility criteria

Patients were recruited from the out-patient clinic, the Department of Oral Medicine, Oral Diagnosis and Periodontology, Faculty of Dentistry, Ain Shams University, and Faculty of Oral and Dental Medicine, Future University.

Both genders between the ages of 25 and 60 years old, systemically free based on a structured medical questionnaire, and with clinically and histologically confirmed painful erosive or atrophic OLP according to the American Academy of oral and maxillofacial pathology [18] and a minimum clinical score of 2 were included. The exclusion criteria included; pregnancy, breast-feeding, smoking, history of drug-induced lichenoid lesions, loss of pliability or flexibility in the tissues involved by the oral lesions of lichen planus, potential treatment of OLP for less than 2 weeks by topical and 4 weeks’ systemic therapy before study, and known hypersensitivity or severe adverse effects to the treatment drugs or to any ingredient of their preparation [16].

### Patients’ grouping, treatment protocol, and interventions

At baseline, all patients had full mouth rehabilitation including: instructions in self-performed plaque control measures, full mouth supra and subgingival scaling and removal of any local irritating factors (fractured teeth, poor restorations, or fixed or removable prosthodontics) if needed. For the purpose of standardization, out of multiple oral lesions, a "marker lesion" was recorded and specified for each patient, being the most severe and painful lesion (minimum clinical score of 2) in the buccal mucosa at baseline.

**Fig. 1:**
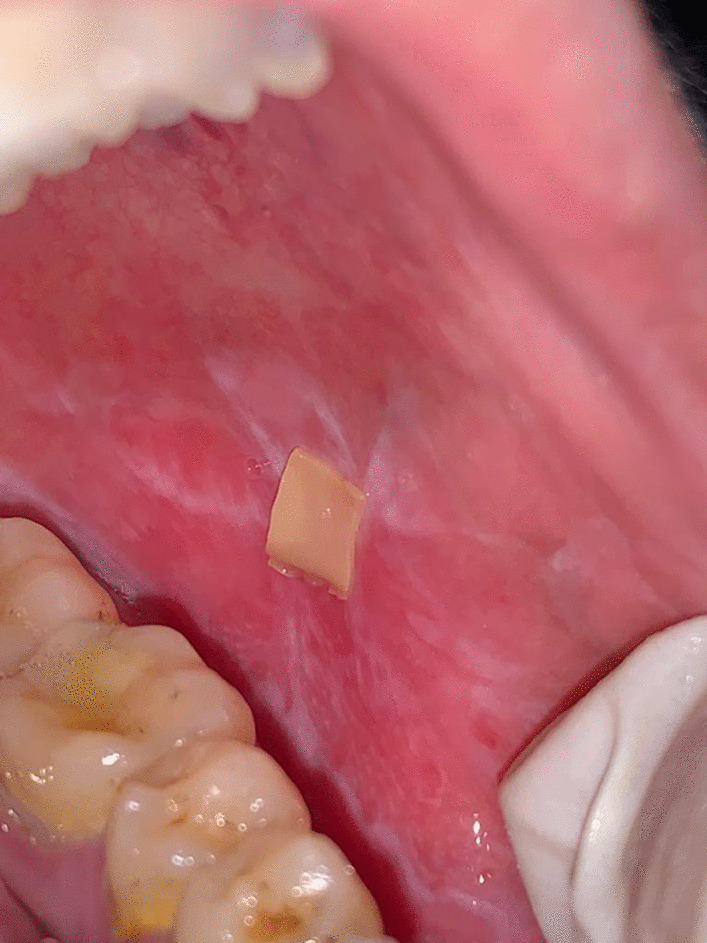
Tacrolimus mucoadhesive patch applied to the marker lesion on the buccal mucosa in Group I patient

Eligible patients were then randomly assigned into three study groups;

*Group I (TAC patch)* (n = 10) patients were instructed to apply a tacrolimus buccal mucoadhesive patch on the marker lesion as shown in Fig. 1 twice daily for 8 weeks containing [10% Tacrolimus (0.1% weight/weight [w/w]), Chitosan 2% (weight/volume [w/v]), 3% w/v hydroxypropyl methyl cellulose (HPMC) in addition to propylene glycol, acetic acid and citric acid in 100 ml distilled water [15] (Nano-Gate Company, Mokattam, Cairo, Egypt). Patients were instructed to apply slight pressure on the entire surface of the patch at the time of application either using their finger or tongue, to refrain from bringing their teeth into contact with the patch, to avoid chewing or excessive jaw movements, and to avoid eating or drinking for at least 1 h following application of the patches [19].

*Group II (TAC gel)* (n = 10) patients received topical tacrolimus gel on marker lesion four time daily for 8 weeks containing [Tacrolimus 0.1% weight/weight [w/w]) to 2% w/w sodium Carboxy methylcellulose (CMC) as gelling agent dispersed in 25 ml of distilled water 0.1%] [20] 4 times/day.

*Group III (TRI. gel)* (n = 10) received topical 0.1% triamcinolone acetonide in the same gel carrier used with tacrolimus gel 4 times/day. Miconazole 2% topical antifungal (Miconaz® oral gel: Miconazole 2 g per 100 gm). (Medical Union Pharmaceuticals—MUP—Egypt) was applied only in the 4th week and 8th week of the treatment period to avoid secondary candidiasis in this group [16].

All patients in gel groups were instructed to apply a thin layer (½ ml) guided by graduation on plastic syringes loaded with the gel medication using a finger or cotton tip applicator on dried lesions after meals, considering not to eat, drink, or speak for at least 1 h afterward [16]. Patients in all groups were also instructed to apply the same intervention to other symptomatic lesions whenever needed.

### Clinical & histological assessment

Patients were evaluated in 5 visits over the course of the 12-week study: at baseline (after 7 days from diagnostic biopsy), at the end of the 2nd, 4th, and 8th weeks of treatment, and after 4 weeks of treatment cessation (12th week).

The clinical effectiveness was evaluated using the clinical scoring (CS), in which “0” represented no lesion/normal mucosa; “1” mild white striae/no erythematous area, “2”white striae with an atrophic area less than 1 cm^2^, “3” white striae with an atrophic area more than 1 cm^2^, “4” white striae with erosive area less than 1 cm^2^, and “5” white striae with an erosive area more than 1 cm^2^ [16].

Photographs of the oral marker lesion for each patient and in every visit were taken using the same digital camera with the same settings (Panasonic Lumix DMC-FH1, resolution 4000 × 3000, 12 mega pixels, 5× optical zoom, with auto macro focus range of 5 cm, Panasonic. Co.). Digitalized images were taken for the analysis using (Adobe Photoshop CS3 Extended version 10.0. Adobe Systems Inc. USA). The total areas of atrophy (TAA) in each marker lesion were outlined and measured in pixels, then transformed into actual surface areas in mm^2^ using the reference true length. Measurements were recorded the same way in each visit and the data was tabulated for analysis [16].

Patients also ranked the severity of pain and burning sensation on a 100-mm visual analogue scale (VAS) [21]. Any unwanted side effects were reported and patients were examined generally for any abnormal vital signs or any alteration in the appearance of mucosa at each visit.

On two occasions, a punch biopsy using a sterile single-use biopsy punch (Kai industries co., ltd. Japan) for approximately 4 mm in width and 5 mm in depth was carried out at the first evaluation visit and at the same site after 8 weeks of treatment. The biopsy site within the marker lesion for each patient was selected so as to avoid areas completely denuded of epithelium and to include keratotic areas and part of normal looking mucosa.

Specimens were fixed in 10% buffered formalin solution, dehydrated, and embedded in paraffin blocks for hematoxylin and eosin staining for initial diagnosis. Other sections were prepared on positively charged glass slides; in summary, immunohistochemical staining is performed as follows: The formalin-fixed, paraffin-embedded specimens in the other block are subsequently cut into 3-µm-thick sections using a tissue microtome, and mounted on aminosilane-coated positively charged glass slides. Sections were then deparaffinized and rehydrated with a graded ethanol series before being immunostained with a labelled streptavidin–biotin immunohistochemistry assay (Lab Vision Corporation, United Kingdom, Ultra-Vision Detection System Anti-Polyvalent, HRP/DAB (Ready-To-Use) TP-015-HD). Photomicrographs of tissue sections were captured at a magnification of 20× using a digital video camera (Canon EOS 650D, Japan) mounted on a light microscope (BX60, Olympus, Japan). For immune-stained sections in each positive section at least four microscopic fields showing the highest immunopositivity were selected (nuclear staining of any intensity was considered positive, and blue nuclear staining was considered negative). Photomicrographs were then transferred to the computer system for analysis for both the number and localization of Caspase-3 immuno-positive cells in the lining epithelium, and in the underlying connective tissue using image analysis software (Image J, 1.41a, NIH, USA). The area fraction of the positive cells, then the mean area fraction (MAF) representing the mean of percentage of immunopositive area to the total area of the four microscopic field, lymphocytic cell counts for selected fields were also calculated automatically.

### Statistical analysis

The collected data was tabulated in an Excel sheet and statistically analyzed using SPSS Version 18 (SPSS Inc., Chicago, IL, USA). Data was expressed in terms of mean and standard deviation [mean (SD)] or frequencies and percentages [n (%)] when appropriate. The data was explored for normality by checking the data distribution and using the Kolmogorov–Smirnov and Shapiro–Wilk tests. Comparisons between groups with respect to normally distributed numeric variables were compared by a one-way analysis of variance (ANOVA) test, followed by a Tukey’s post hoc test when ANOVA revealed a significant difference. A comparison between different observations was performed using the repeated measure ANOVA test. Qualitative variables were expressed as numbers and percentages and were compared using the chi square test. A Pearson’s correlation was also performed. A *P*-value of less than 0.05 was considered statistically significant for all variables.

## Results

**Fig. 2:**
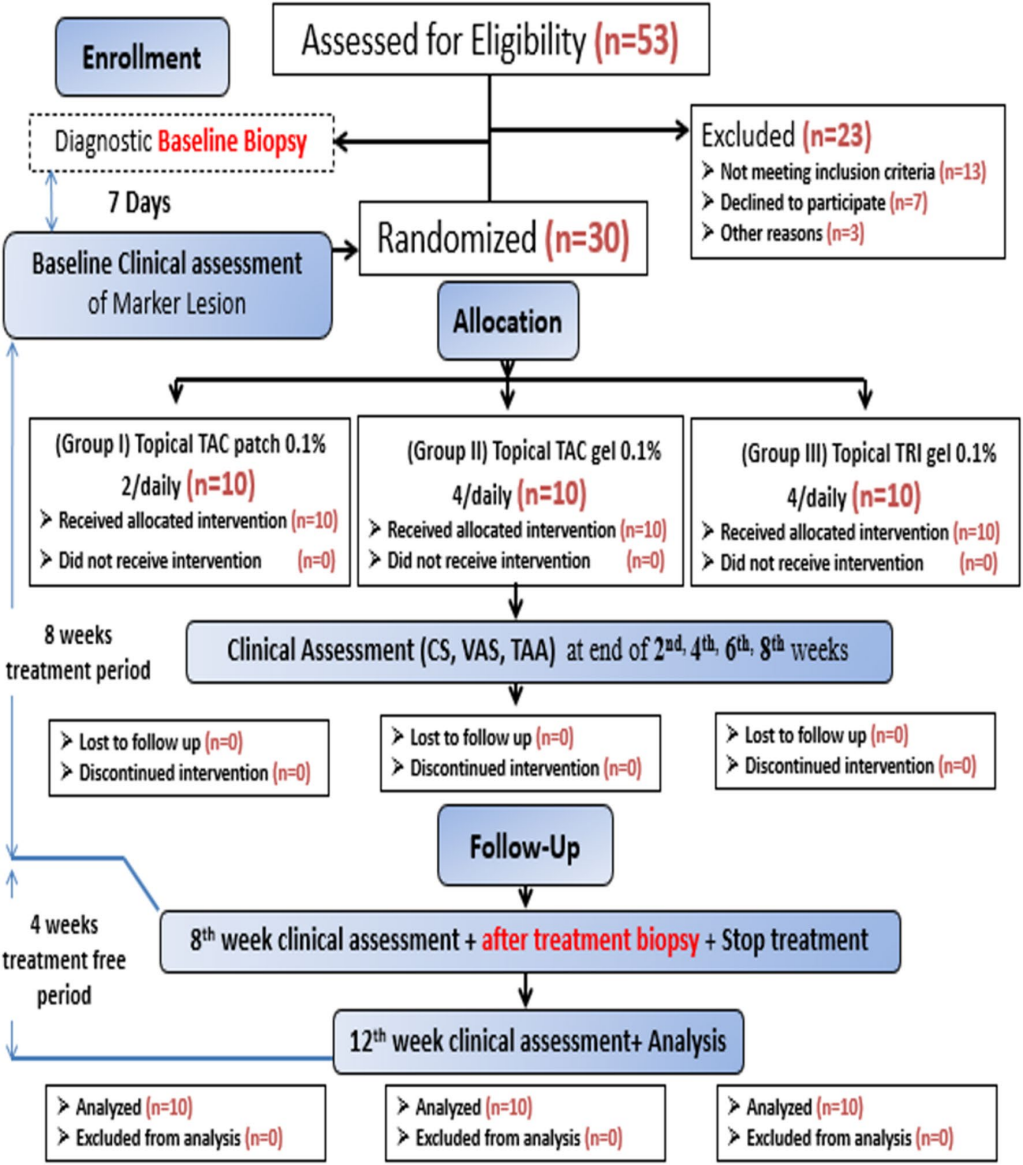
A study CONSORT flow diagram illustrating patients’ enrolment and outcomes assessment

A total of fifty-three patients were examined for eligibility for this study between January 2019 and December 2021. Thirty patients of them [27 females (90%) and 3 males (10%)] were diagnosed both clinically and histopathological with oral lichen planus and met the eligibility criteria to be randomly classified into the three study groups. All patients were committed to the treatment protocol throughout the twelve weeks of the study period with no dropouts as demonstrated in the study flow diagram Fig. 2. The baseline demographics of patients enrolled in the study, as well as clinical characteristics of OLP, including disease duration, lesion type, and frequency of exacerbations, are demonstrated in Table 1.

**Table 1:** The baseline demographic and clinical characteristics of oral lichen planus (OLP) patients enrolled in the study

Group	Age (year) Mean (SD)	Gender Male/female [n (%)]	Disease duration (month) mean (SD)	Clinical type erosive/atrophic [n (%)]
Group I (*n* = 10) [tacrolimus patch]	55.8 (11.88)^a^	0/10 (100%)	5.7 (3.02)	3 (30%)/7 (70%)
Group II (*n* = 10) [tacrolimus Gel]	48.6 (11.62)^a^	2 (20%)/8 (80%)	6.9 (4.43)	5 (50%)/5 (50%)
Group III (*n* = 10) [corticosteroid gel]	41.1 (8.97)^b^	1 (10%)/9 (90%)	3.86 (3.29)	4 (40%)/6 (60%)
*P*-value	0.02*	0.329	0.188	0.659

Values are given as mean (SD), *Significant at p ≤ 0.05 compared to the other study group (Chi square test). Tukey’s post hoc test: within the same comparison, means sharing the same superscript letter are not significantly different

All patients in Group I reported no adverse effects from the tacrolimus patch and showed great compliance compared to other groups in the study. While the most common reported adverse effects were mild burning sensations in 4 patients (40%) in Group I and II in the 1st week and the symptoms disappeared without any medication at the beginning of the 2nd week, there were no complaints of gag reflex or incompliance from any patient.

**Fig. 3:**
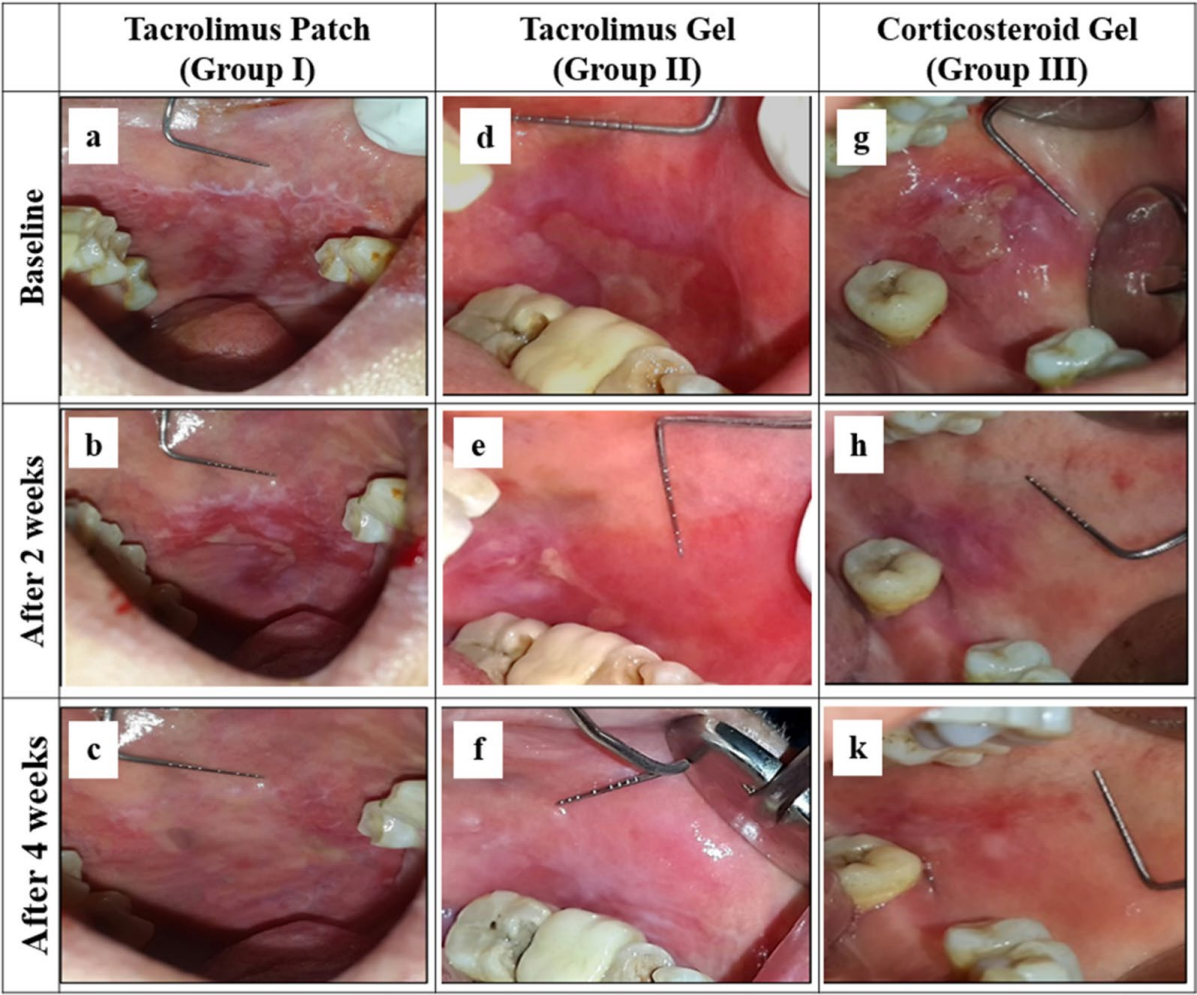
Photographs of erosive and atrophic oral lichen planus marker lesions in the buccal mucosa in 3 patients from Group I (**a**), Group II (**d**), and Group III (**g**), at baseline. Reduction in lesion size and severity by topical Tacrolimus patch (**b**), and slight reduction in ulcer size and extension with topical application of Tacrolimus gel (**e**), and Triamcinolone Acteonid gel (**h**), after 2 weeks’ treatment. Complete healing of oral lesions treated with Tacrolimus patch (**c**), and marked signs of healing of oral lesions from the same patients treated by topical tacrolimus gel (**f**), and corticosteroid gel (**k**), after 4 weeks’ treatment

The Tacrolimus patch resulted in a higher reduction in CS and VAS within the first two weeks than the other groups, and the same was observed in the whole treatment and follow-up periods as shown in Table 2. Clinical changes in marker lesions with treatment in all study groups were also observed in photographs in Fig. 3.

**Table 2:** Comparison between groups regarding percentage change in clinical scoring (CS), visual analogue scale (VAS) and total atrophic area (TAA) in different study intervals

% Change in parameters	Group I	Group II	Group III	*P*-value
*CS [mean (SD)]*				
Base-2w	− 14.00 (15.54)	− 9.33 (12.65)	− 8.67 (14.42)	0.664
2w–4w	− 10.00 (21.08)	− 5 (15.8)	− 21 (27.3)	0.732
4w–8w	− 10.00 (21.08)	− 20.00 (25.82)	0 (0)	0.085
Base-8w	− 32.00 (24.25)	− 31.33 ± 28.07	− 28.67 (26.26) 0.956	
8w–12w	− 70.00 (25.82)	− 45.00 (43.78)	− 30 (42.16) 0.078	
*VAS [mean (SD)]*				
Base-2w	− 70.21 (15.82)^a^	− 66.22 (18.66)^a^	− 18.27 (8.88)^b^	0.000*
Base-4w	− 77.17 (32.36)^a^	− 80.48 (24.03)^a^	− 26.20 (11.12)^b^	0.000*
Base-8w	− 100.00 (0.00)	− 81.67 (29.11)	− 62.51(41.60)	0.144
Base-8w	− 100.00 (0.00)	− 97.64 (4.99)	− 75.83 (27.47)	0.576
8w–12w	Not computed	− 100.00 ± 0	− 90.00 (22.36)	0.004*
Base-2w	− 22.56 (8.00)^a^	− 30.16 (12.74)^a^	− 16.08 (8.36)^b^	0.014*
*TAA [mean (SD)*				
Base-4w	− 28.46 (10.39)^a^	− 28.54 (11.47)^a^	− 14.64 (6.19)^b^	0.004*
Base-8w	− 32.99 (14.86)	− 36.11 (12.68)	− 22.72 (18.35)	0.148
Base-8w	− 63.23 (9.52)^a^	− 67.75 (11.68)^a^	− 43.97 (15.9)^b^	0.001*
8w–12w	− 62.41 (2.64)^a^	− 43.33 (19.9)^a^	− 29.6 (7.92)^b^	0.012*

Values are given as mean (SD), *Significant at p ≤ 0.05 compared to the other study group (ANOVA) test, followed by Tukey’s post hoc: within the same comparison, means sharing the same superscript letter are not significantly different

**Fig. 4:**
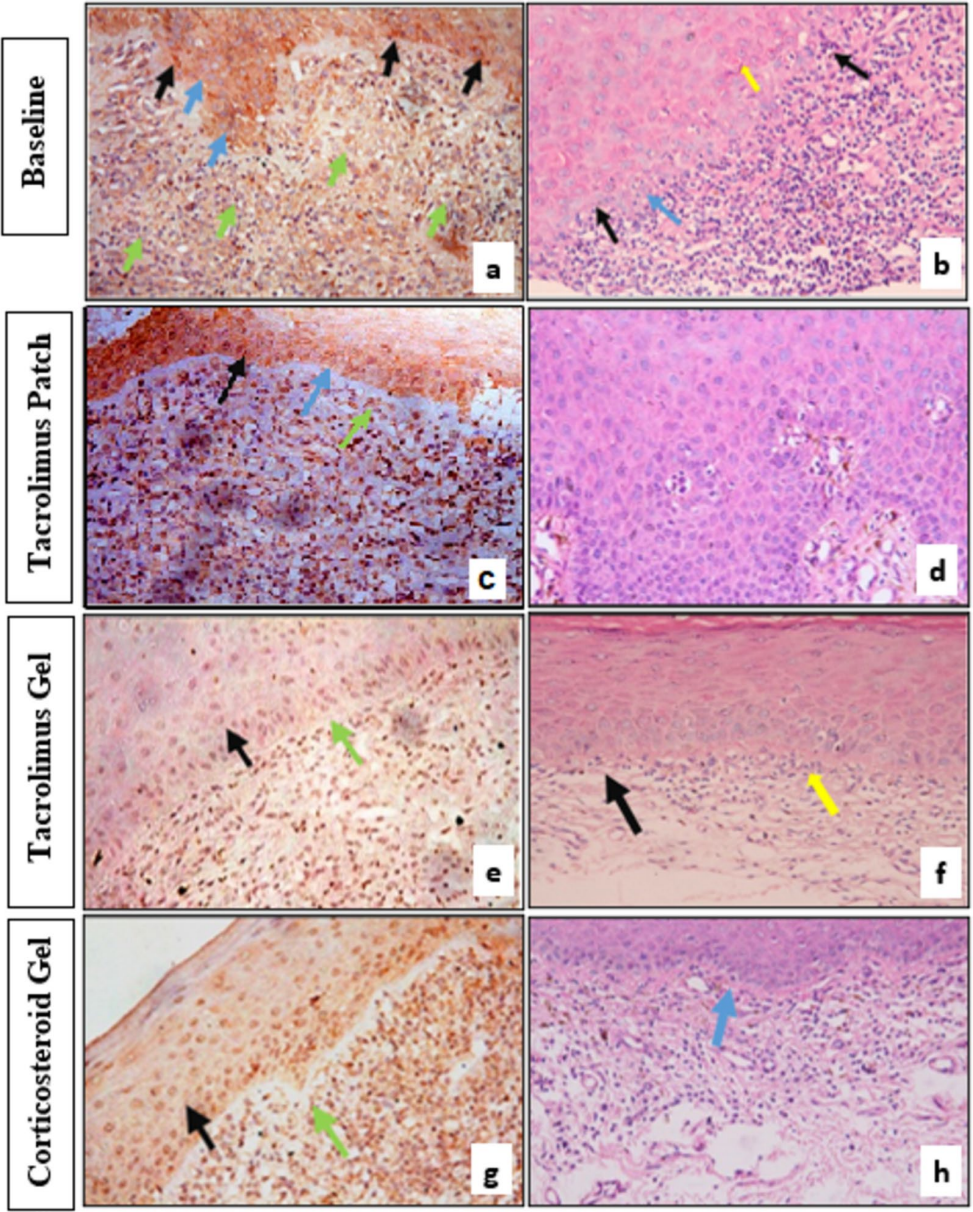
**a** Photomicrograph of OLP specimen at base line showing nuclear and cytoplasmic immunopositivity of some of the epithelial cells (black arrow) while other cells exhibited cytoplasmic immunopositivity and immunonegative nuclei (blue arrow). The connective tissue cells were found to be more frequently immune negative (green arrow). (caspase 3 × 40). **b** Photomicrograph of OLP specimen at base line showing dead keratinocytes (civatte bodies) (yellow arrow), vacuoles in the basal cell layer (blue arrow). The epithelial connective tissue junction obscured by lymphocytes (black arrow). (H&E ×40). **c** Photomicrograph of OLP specimen after treatment with tacrolimus patch, showing epithelial cells with cytoplasmic immunopositivity (black arrow), while the majority of epithelial cells nuclei were immunonegative (blue arrow). Some connective tissue cells were immunopostive (green arrow). (Group I) (caspase 3 × 20). **d** Photomicrograph of one of the OLP specimens after treatment with tacrolimus patch, showing hyperplastic epithelium. Note the bland appearance of the hyperplastic epithelium. Group I (H&E ×40). **e** Photomicrograph of OLP specimen after treatment with tacrolimus gel, exhibiting mild nuclear and cytoplasmic immunopositivity in some of the epithelial cells (black arrow) and most of the connective tissue cells (green arrow). (Group II) (caspase 3 × 20). **f** Photomicrograph of OLP specimen after treatment with tacrolimus gel showing relatively scant lymphocytes (yellow arrow), evident epithelial-connective tissue junction (black arrow). Group II (H&E ×20). **g** Photomicrograph of OLP specimen at base line showing nuclear and cytoplasmic immunopositivity of the epithelial cells (black arrow). Most of the connective tissue demonstrated immunopostive reaction (green arrow). (caspase 3 × 20) (Group III). **h** Photomicrograph of OLP specimen after treatment showing few lymphocytes, evident epithelial-connective tissue junction notes some basal epithelial cells exhibiting vacuolation (blue arrow). Group III (H&E ×20)

Photomicrographs of OLP H/E-stained section after treatment showed scant subepithelial infiltrating T-lymphocytes in the three groups. While the changes in Caspase-3 expression as a marker of apoptosis in the study groups following treatment both within the epithelium and connective tissue are demonstrated in photomicrographs in Fig. 4. A significant reduction in Caspase 3 expression with tacrolimus patch and gel compared to a significant increase observed with corticosteroid gel within the epithelium. While in connective tissue, Caspase 3 expression increased in all groups but was highest following treatment with corticosteroid gel as shown in Table 3.

**Table 3:** Comparison between groups in mean percentage change in Caspase-3 expression and lymphocytic count in studied groups before and after treatment

Mean percentage change [mean (SD)]	Group I	Group II	Group III	*P*-value
Caspase-3 (connective tissue)	− 25.5014^a^ (18.23)	− 29.9224^a^ (27.36)	− 118.3261^b^ (104.92)	0.004
Caspase-3 (epithelium)	59.2923^a^ (19.63)	58.7310^a^ (16.76)	− 64.2289^b^ (33.11)	0.0001*
Lymphocyte count	64.5492^a^ (7.42)	39.0625^b^ (6.42)	26.8015^c^ (13.45)	0.0001*

Values are given as mean (SD), *Significant at p ≤ 0.05 compared to the other study group, ^#^significant at p ≤ 0.05 compared to base line (ANOVA) test, followed by Tukey’s post hoc: within the same comparison, means sharing the same superscript letter are not significantly different

A significant correlation (p ≤ 0.05) between the percentage change in Caspase expression in the epithelium and the CS (0.475) and TAA (0.494) were noted in the studied groups.

## Discussion

Tacrolimus in an oral patch carrier system was compared to both tacrolimus gel and corticosteroid gel as standard of care for the management of OLP. A placebo patch as a negative control intervention would not have been applicable due to ethical standards for managing symptomatic lesions. Moreover, the patch was applied only 2 times/day because its release profile ranged from 10 to 12 h and the patch disintegration time was 30–45 min with minimal salivary washout compared to oral gel [15]. The previous data were also according to manufacturer characterization performed for both Tacrolimus patch demonstrated in Additional file 1, and for the carrier gel in Additional file 2.

The study demonstrated that both tacrolimus and triamcinolone, regardless of carrier system, showed significant reductions in all clinical parameters after treatment termination. However, there was no significant difference between groups. These results were consistent with other studies comparing corticosteroids with calcineurin inhibitors [10, 13, 22].

Topical application of tacrolimus could initially stimulate sensory neurons through substance P release. This would explain the transient burning or painful sensations documented in tacrolimus groups. However, the calcium influx provoked by the neuron activation probably led to a depletion of phosphatidylinositol 4,5-bisphosphate (PIP2) and subsequent inactivation of the calcium channels [23]. This would explain the analgesic effect which was maintained by close contact and deep penetration associated with the patch, which was consistent with Ahmed et al. 2018 who reported that at the end of 12th week follow-up periods, complete relief of pain and burning sensation with 0.1% tacrolimus gel was 100% [24].

Siponen et al. showed that tacrolimus and triamcinolone acetonide 0.1% ointment had a great reduction in clinical score at 9 weeks of treatment with percent change of 37% and 32% respectively with no statistically significant difference between them [17], which were comparable to our results, which showed CS reduction in tacrolimus patch, tacrolimus gel and triamcinolone acetonide at 8 weeks of treatment with percent change of 32%, 31% and 28% respectively with no significant difference between them. Furthermore, tacrolimus patch in current study induced a reduction in lesion size after 2 months of treatment to 63%, the tacrolimus gel group to 67%, and the triamcinolone acetonide group to 43%, with a significant difference between the study groups and control group. These results were also consistent with Hettiarachi 2017 who reported that mean CS was reduced by 1.18 in the tacrolimus group compared with a reduction of 0.5 in the clobetasol group after 3 weeks of treatment and this difference was considered statistically significant [16].

Tacrolimus patch and gel showed better initial reduction in TAA compared with triamcinolone 0.1% Group III with significant reduction starting from the fourth week till the end of the follow-up period, and these results were consistent with Laeijendecker et al. (2006) who reported that topical tacrolimus 0.1% ointment induced a better initial therapeutic response than triamcinolone acetonide 0.1% ointment. However, relapses occurred frequently within 3–9 weeks of the cessation of treatment [25], but in the current study the patch maintained improvement during the treatment free period, which is probably due to better mucoadhesive properties, longer release profile, and better patient compliance during the treatment period, as well as a higher percentage of penetration enhancer (propylene glycol) than the oral gel.

The previous interpretation was documented in the recent meta-analysis by Zhangci et al. (2022), which reported that there was no difference in clinical resolutions between tacrolimus and corticosteroids and recommended the use of mucoadhesive carrier systems with a sustained release profile for improving the clinical outcomes [26], which were also demonstrated in our study.

Caspase-3 expression decreased in epithelium and increased in connective tissue after treatment in both tacrolimus groups, whereas corticosteroid treatment increased in both epithelium and connective tissue. This could be justified by the fact that, tacrolimus is more selective for lymphocytes, and hence fewer lymphocytes expected to express caspase-3. Furthermore, the anti-inflammatory tacrolimus effect is not exclusively through apoptosis but through inhibition of T-cell activation from the start [27]. Subsequently, as T lymphocytes stopped induction of apoptotic signals to basal cells in the epithelium due to a reduction in their number, a high expression of caspase in the connective tissue is observed. In contrast, corticosteroids have a non-specific induction of apoptosis [27]. Moreover, it should be noted that some cells exhibited caspase-3 gene expression but it was localized in the cytoplasm and not the nucleus. Alongside this pattern of expression, focal areas of epithelial hyperplasia were seen in the studied specimens of these groups because when caspase-3 becomes activated, it then enters into the nucleus to perform its effect. It is implied that nuclear caspase-3 expression may be greater from the functional standpoint of view [28].

Generally, this study showed that topical tacrolimus patch had superior and initial rapid clinical effect with higher patient compliance than tacrolimus and triamcinolone acetonide gel, although statistically insignificant. These findings may be attributed to a reduction in the number of T-lymphocyte cells and caspase-3 expression within the basal epithelium. The study was limited by the short follow-up, precluding the opportunity to evaluate the relapse rate and highlighted the need to assess treatment durability with further clinical trials.

## Supplementary Information

**Additional file 1**. Manufacturer’s characterization of tacrolimus patch.

**Additional file 2.** Manufacturer's characterization of tacrolimus and triamcinolone gel carriers.

## Data Availability

The datasets used in the current study are available from the corresponding author upon request.

## Abbreviations

TAC: Tacrolimus
OLP: Oral lichen planus
HPMC: Hydroxypropyl methylcellulose
CMC: Carboxymethylcellulose
TAA: Total areas of atrophy
WHO: World Health Organization
CS: Clinical scoring
VAS: Visual analogue scale
MAF: Mean area fraction
H&E: Hematoxylin & Eosin
ANOVA: One-way analysis of variance
