# Supplementary material for: Evaluation of muco-adhesive tacrolimus patch on caspase-3 induced apoptosis in oral lichen planus: a randomized clinical trial
Source: BMC Oral Health. 2023 Feb 14;23:99. doi: 10.1186/s12903-023-02803-8 (PMC9930326; doi:10.1186/s12903-023-02803-8)
Supplement: Supplementary file 1 — Additional file 1. Manufacturer’s characterization of tacrolimus patch. [file 12903_2023_2803_MOESM1_ESM.pdf]

Tacrolimus loaded Chitosan patch

Preparation Method:

Materials:

Chitosan low molecular wight, DD = 85%, HPMC and propylene glycol were purchased from (Loba Chemie India), glacial acetic acid and citric acid were purchased from (Chem-lab, Belgium)

Preparation of Tacrolimus loaded Chitosan patch

Chitosan 2% weight/volume and HPMC 3% weight/volume were dissolved in 1% dil.acetic acid and distilled water respectively and these two solutions were mixed together. Citric acid dissolved in distilled water and propylene glycol was added to the above solution mixture. To this the required quantity of Tacrolimus was added by dissolving the drug in a small quantity of ethanol with final concentration 0.1%w/w. The mixture was kept under magnetic stirrer for 5 minutes. Then it was casted to the petridish and kept for drying at room temperature.

Weight and patch thickness

The assessment of weight and patch thickness were completed on randomly selected patches from three patches. For mass determination, patches were weighed on an electronic digital balance

and its thickness measured at 3 different randomly determined points using Vernier calipers, **(Zhang et al, 2018)**.

Surface PH

Was determined by dissolving the patches in dH2o for 5 minutes and measurements recorded using a PH meter, **(Colley,2018)**.

Folding indurance

Was determined by repeatedly folding a small strip of the patch at a same place till it broke. The number of the times the patch could be folded at the same place without breaking gives the value of folding indurance which indicates the brittlness of the patch.

Folding indurance was determined in triplicate and the mean value was calculated, **(Colley,2018)**.

Percent Elongation

The initial lengths of the patches were measured and then stretched to a lesser extent and final length was noted, **(Semalty et al, 2008)**.

Drug Content

A patch was placed in a beaker containing 5 ml phosphate buffer (PH 6.8) and 5 ml alchol. Medium was stirred for proper dissolution on orbital shaker for 4 hours, then the content was filtered using whatman filter paper and the filtrate sample was analysed by UV spectrophotometer at 297nm, **(Rathi et al, 2011)**.

Patch softening upon storage

Patches were stored in desiccators for 48 hours and softening was determined, **(Zhang et al, 2018)**.

Disintigration Time

Is the time at which a patch breaks when brought in contact with water or saliva. Patches were immersed in a beaker containing 25ml phosphate buffer 6.8.

It was swirled at every 10 seconds and the time at which the patch started to break was recorded, **(Ashwathy et al, 2019)**.

Release profile:

Were performed using USP paddle type apparatus. The studies were carried out at 37 C with stirring speed of 100 rpm in 900 ml phosphate buffer 6.8. 5 ml of samples were withdrawn at predetermined time intervals of 0.5, 1, 1.5, 2, 2.5, 3, 3.5, 4, 4.5, 5, 5.5, 6 hours and replaced with the same value of buffer. The samples were collected and the concentration was determined at 297nm using UV, **(Rathi et al, 2011)**.

Results

Weight and patch thickness

Tacrolimus patches were recorded to have an average weight 67.4, 59.0 and 54.0 mg, weight differences were not significant.

Average thickness of the patches were 0.51, 0.46 and 0.46, the differences were not significant.

Surface PH

The value for surface PH were between 6.0 and 6.1 for the different tacrolimus patches and the difference were not significant.

Folding Indurance

Varied from 84-89 and it increase with the elevation in the concentration of HPMC K and M proportion.

Percent Elongation

was found to be 50%.

Drug Content

The results ranged from 70.93% to 67.48%.

Film softening upon storage

No softening occurred to the patches upon storage.

Disintigration Time

For the tacrolimus patches, disintigratiom time was found to be 30-45 minutes.

Release Profile.

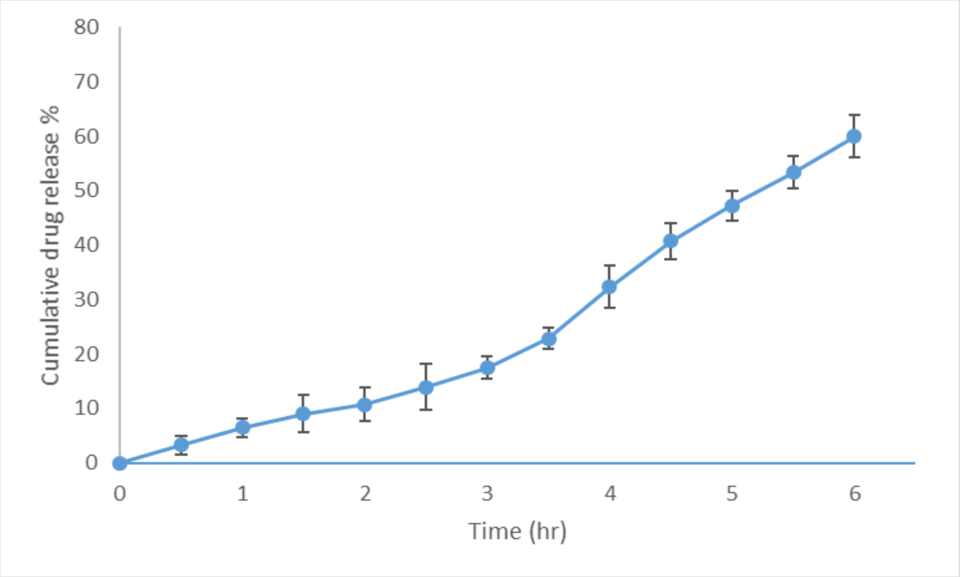

In-vitro release of Tacrolimus patch in phosphate buffer saline pH 6.8 at 297 nm
