# Supplementary material for: Evaluation of muco-adhesive tacrolimus patch on caspase-3 induced apoptosis in oral lichen planus: a randomized clinical trial
Source: BMC Oral Health. 2023 Feb 14;23:99. doi: 10.1186/s12903-023-02803-8 (PMC9930326; doi:10.1186/s12903-023-02803-8)
Supplement: Supplementary file 2 — Additional file 2. Manufacturer's characterization of tacrolimus and triamcinolone gel carriers. [file 12903_2023_2803_MOESM2_ESM.pdf]

# Preparation of Tacrolimus and Triamcinolone acetonide Oral gels

0.1% Tacrolimus gel and 0.1 % triamcinolone acetonide gel were prepared using 2 % weight/weight sodium Carboxy methylcellulose (CMC) as gelling agent as described previously with little modification **(Suryakumari et al 2019)**. The weighed quantity of CMC sprinkled slowly on the surface of purified water under continuous mechanical stirring at 600 rpm to get homogenous dispersion. Methyl paraben and propyl paraben were added to the gel as preservative with continuous stirring till it got dispersed in gel completely.

Accurately weighed amount of drug either Tacrolimus or triamcinolone acetonide was dissolved in ethanol. The drug solution was added slowly to the gel to give final concentration 0.1% Tacrolimus or 0.1% of triamcinolone acetonide with the help of stirrer to obtain homogenous dispersion of gel **(Lakshmi et al,2011)**.

## Characterization of oral gel

### 1) Determination of pH

The pH of prepared gels was detected using a digital pH meter (Mettler Toledo MP 220, Greifensee, Switzerland) at 25 °C in triplicate. 2.5 gm of gel was accurately weighed and dispersed in 25 ml of distilled water, then the pH was determined **(Harsha et al,2017)**.

### 2) Determination of Viscosity

Viscosity of Tacrolimus gel as well as triamcinolone acetonide gel was evaluated using Brookfield viscometer (DV-II, LV model, Brookfield, USA). The viscosity was determined (n=3) The measurement of the viscosity was done (n=3) at room temperature

(25°C±1°C) at different rotational speeds from 0.5-20 rpm with a torque of near to 100%. The samples were equilibrated for 10 min prior to the analysis **(Lakshmi, et al 2011)**.

**3) Determination of Spreadability**

One of the requisites for a gel preparation to meet the ideal qualities is a wide spreadability. It is evaluated by the extent of area to which gel immediately spreads at site of application. The spreadability was determined by applying 0.5 g gel within a pre-marked circular area of 1 cm diameter on a glass plate with specifications of 5 mm thickness and 15 $cm^2$ area. And another glass plate with equal dimensions was placed on it; ensure that entrapment of the air bubbles avoided between two slides. Standard weight of 500 g was placed on the upper glass plate for 5 min to spread the gel uniformly. Higher the area of the gel spread over the plate is an indicator of efficient spreadability **(Harsha et al, 2017)**.

**4) Determination of drug release profile**

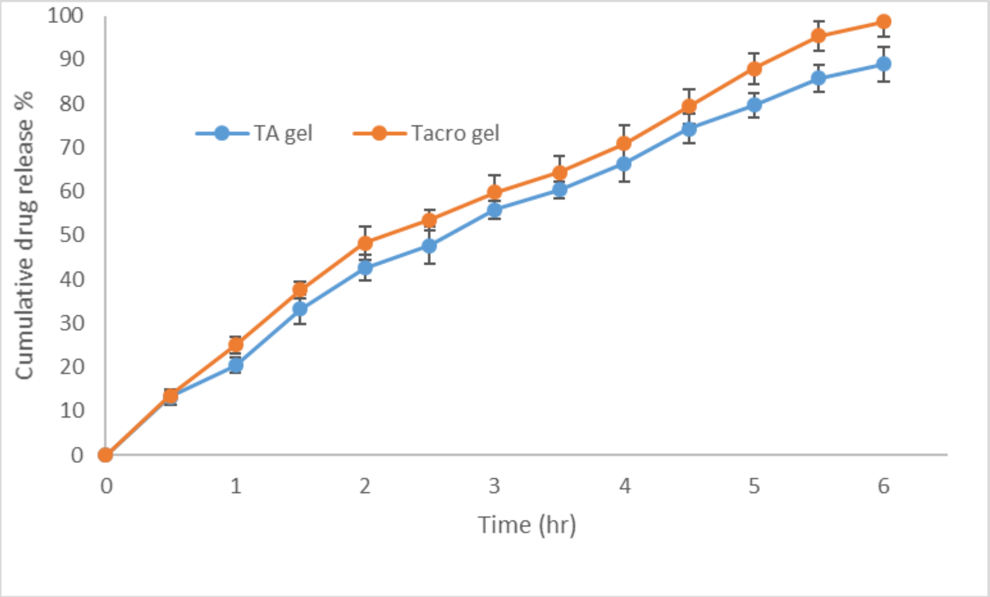


**In-vitro release of Tacrolimus gel and triamcinolone acetonide gel in phosphate buffer saline pH 6.8 at 297nm and 240 nm.**

## Results

### 1) Determination of pH

The pH was found to be 6.3±0.12 and 6.5±0.16 for Tacrolimus and triamcinolone acetonide gel respectively, which is consider suitable with oral cavity

### 2) Determination of Viscosity

Viscosity is an important rheological parameter involved in using gel. Since higher viscosity hinders the instillation and lower viscosity cause drainage. So, an ideal viscosity gel is necessary to show effective delivery. Our produced gel showed viscosity of 189036 cps and 190025cps for Tacrolimus and triamcinolone acetonide gel respectively, which is consider suitable

### 3) Determination of Spreadability

Spreadability is a vital feature of semisolid dosage forms which influence the ease of administration and patient compliance. Ideal gel will spread in a short duration which ultimately enhances the ease of application. Spreadability is calculated by the change in the diameter of earlier drawn circle (1 cm) by the application of weight. Tacrolimus gel exhibited 8.2 cm and triamcinolone acetonide gel exhibited 8.4 cm which reflects good spreadibility.

**Dr. Asmaa M. Elbakry [*Asmaa M. Elbakry*]**

Assistant Professor of Pharmaceutics and Pharmaceutical Technology,

Faculty of Pharmacy, Al Azhar University, Cairo, Egypt.
